# Comparison of single-cell long-read and short-read transcriptome sequencing via cDNA molecule matching: quality evaluation of the MAS-ISO-seq approach

**DOI:** 10.1093/nargab/lqaf089

**Published:** 2025-07-04

**Authors:** Natalia Zajac, Qin Zhang, Anna Bratus-Neuenschwander, Weihong Qi, Hella Anna Bolck, Tülay Karakulak, Tamara Carrasco Oltra, Holger Moch, Abdullah Kahraman, Hubert Rehrauer

**Affiliations:** Functional Genomics Center Zurich, ETH Zurich and University of Zurich, 8057 Zurich, Switzerland; Swiss Institute of Bioinformatics, Amphipôle, Quartier UNIL-Sorge, 1015 Lausanne, Switzerland; Functional Genomics Center Zurich, ETH Zurich and University of Zurich, 8057 Zurich, Switzerland; Functional Genomics Center Zurich, ETH Zurich and University of Zurich, 8057 Zurich, Switzerland; Functional Genomics Center Zurich, ETH Zurich and University of Zurich, 8057 Zurich, Switzerland; Swiss Institute of Bioinformatics, Amphipôle, Quartier UNIL-Sorge, 1015 Lausanne, Switzerland; Department of Pathology and Molecular Pathology, University Hospital Zurich, Schmelzbergstrasse 12, 8091 Zurich, Switzerland; Centre for AI, School of Engineering, Zurich University of Applied Sciences (ZHAW), Technikumstrasse 71, 8400Winterthur, Switzerland; Swiss Institute of Bioinformatics, Amphipôle, Quartier UNIL-Sorge, 1015 Lausanne, Switzerland; Department of Pathology and Molecular Pathology, University Hospital Zurich, Schmelzbergstrasse 12, 8091 Zurich, Switzerland; Institute of Molecular Life Sciences, University of Zurich, Winterthurerstrasse 190, 8057 Zurich, Switzerland; Functional Genomics Center Zurich, ETH Zurich and University of Zurich, 8057 Zurich, Switzerland; Department of Pathology and Molecular Pathology, University Hospital Zurich, Schmelzbergstrasse 12, 8091 Zurich, Switzerland; Institute of Molecular Life Sciences, University of Zurich, Winterthurerstrasse 190, 8057 Zurich, Switzerland; Swiss Institute of Bioinformatics, Amphipôle, Quartier UNIL-Sorge, 1015 Lausanne, Switzerland; School for Life Sciences, Institute for Chemistry and Bioanalytics, University of Applied Sciences Northwestern Switzerland, Hofackerstrasse 30, 4132 Muttenz, Switzerland; Functional Genomics Center Zurich, ETH Zurich and University of Zurich, 8057 Zurich, Switzerland; Swiss Institute of Bioinformatics, Amphipôle, Quartier UNIL-Sorge, 1015 Lausanne, Switzerland

## Abstract

Single-cell RNA sequencing is used for profiling gene expression differences between cells. It can be performed with short reads, which provide high-throughput and high-quality information at the gene level, or with long reads, which provide isoform resolution via preserving full-length transcripts. It is, however, unclear how comparable the data is between the two methods. We investigate the types of bias introduced at the library preparation and the bioinformatic processing steps on the transcripts recovered from long- and short-read sequencing, and the effects of filtering, enabled by sequencing of full-length transcripts, on gene expression. For each sample, we sequenced the same 10x Genomics 3′ complementary DNA (cDNA) using Illumina short reads and Pacific Biosciences long reads and cross-compared each molecule matched through cell barcode and unique molecular identifier. We find that both methods render highly comparable results and recover a large proportion of cells and transcripts. However, platform-dependent cDNA library processing and data analysis steps introduce biases. Short-read sequencing provided higher sequencing depth, but long-read sequencing allowed for retaining transcripts shorter than 500 bp and for removal of degraded cDNA contaminated by template switching oligos. Filtering of artefacts, identifiable only from full-length transcripts, reduces gene count correlation between the two methods.

## Introduction

Single-cell RNA sequencing (scRNA-seq) is a method for gene expression profiling at a resolution of individual cells [1, 2]. Rapid developments in the library protocols in this field have expanded the collected data with multiple modalities from single cells, including chromatin accessibility (ATAC-seq) [1–4], surface protein abundance (CITE-seq) [1, 5, 6], adaptive immune receptor repertoire [7], nucleosome occupancy [5, 8, 9], or spatial information [10]. The data have shed light on the understanding of the heterogeneity in cell types and subtypes [11–13], cell states [14, 15], cell fate [16, 17], and cell-to-cell interactions [18, 19], not only at the level of a single organism but also at the inter-organismal level, e.g. of the host and its pathogens [20].

Short-read sequencing has been the basis for scRNA-seq and for all the above-mentioned applications, but this method comes with its limitations—the transcripts are only partially sequenced either from the 3′ or 5′ end, and the reads are fixed to an exact same length. Long-read sequencing, on the other hand, has allowed for full-length transcript sequencing [21], in consequence providing information on isoform expression as well as on the single nucleotide and structural variants along the whole length of the transcripts [21–23]. Alternative splicing, differential isoform expression, and variation in sequence identity between isoforms have been shown to have a significant contribution to understanding the complexity of systems classically addressed with scRNA-seq [24–26].

Historically, long-read data have often been coupled with short-read data due to lacking in coverage (mostly concerning Pacific Biosciences, PacBio, data) or due to lower read accuracy (mostly concerning Oxford Nanopore Technologies, ONT, data) [2, 27]. Recent developments in both PacBio and ONT technologies, including improvements in throughput and read accuracy, as well as significant advances in software for processing long-read data have allowed the decoupling of long- and short-read approaches. For example, increase in throughput has been enabled by the multiplexed array isoform sequencing method (MAS-ISO-seq) offered by PacBio, released in October 2022 and now relabelled as Kinnex full-length RNA sequencing, which consists of concatenating full-length transcripts into longer fragments (with average size of 10–15 kb) that can then be sequenced on their instruments [28] (PacBio Application note—MAS-Seq for single-cell isoform sequencing, https://www.pacb.com/wp-content/uploads/Application-note-MAS-Seq-for-single-cell-isoform-sequencing.pdf, accessed March 2024). Each fragment thus consists of an average of 16 transcripts instead of 1. After sequencing the reads can then be bioinformatically broken down to the original transcripts. The tools that have been instrumental in bridging the gap and improving the applicability of long-read technologies include software such as Flexiplex [29], BLAZE [30], Sockeye (https://github.com/jang1563/sockeye), Scywalker [31], SQANTI3 [32, 33], or workflows like wf-single-cell (ONT) (https://github.com/epi2me-labs/wf-single-cell).

The use of long reads without an addition of short reads can be more cost- and time-efficient. However, questions arise on the comparability of the two sequencing methods. In this paper, we address two main questions for those concerned about the impact of the differences between the short- and long-read sequencing on the obtained results: (i) What are the types of bias introduced at the platform-specific complementary DNA (cDNA) library processing steps or the bioinformatic data analysis steps on the transcripts recovered from long- and short-read sequencing, and (ii) Does the difference in recovered transcripts and the presence of the isoform information alter the conclusions on gene expression.

We sequenced four samples of patient-derived organoid cells of clear cell renal cell carcinoma (ccRCC) and one sample of patient-derived metastasis-free kidney organoid cells using Illumina NovaSeq 6000, a short-read sequencing platform, and PacBio Sequel IIe, a long-read sequencing platform. Both the short- and the long-read sequencing were done from the same 10x Genomics 3′ cDNA, with each cDNA fragment being tagged with a cell barcode and a unique molecular identifier (UMI). We then carried out a per-molecule comparison of the reads mapped to the human genome, and we compared gene count matrices generated with state-of-the-art bioinformatic pipelines. Despite short reads providing higher coverage and generally recovering more UMIs per cell, we find the data from both methods to be highly comparable and render corresponding results for ccRCC-relevant genes. However, sequencing platform-specific library preparation and data analysis steps do introduce biases along the way impacting the recovered cells and transcripts and, in consequence, gene expression results. MAS-ISO-seq library preparation allows for retaining transcripts shorter than 500 bp and for removal of a large proportion of truncated cDNA contaminated by template switching oligos (TSO). PacBio’s single-cell Iso-Seq processing pipeline applies more stringent filtering permitted by identification of artefacts from full-length transcripts.

## Materials and methods

### Generation and characterization of ccRCC patient-derived organoid samples

The Department of Pathology and Molecular Pathology at University Hospital Zürich made available the patient tissue samples. They were collected and biobanked according to previously described procedures [34]. The study was approved by the local Ethics Committee (BASEC# 201 9-01 959) and in agreement with Swiss law (Swiss Human Research Act). All patients gave written consent. Organoids were established as previously described [35]. The details of the methods are described in [36].

### Library preparation

#### 10x Genomics single-cell full-length cDNA synthesis

The samples were analysed using the 10x Genomics Chromium platform [37]. Library preparation was conducted following the 10x Genomics Chromium Single Cell 3′ Reagent Kits User Guide (v3.1 Chemistry Dual Index). Organoids were dissociated, washed to eliminate debris, clumps, and contaminants, and resuspended in a 1× phosphate buffered saline/0.04% bovine serum albumin solution at a concentration of 500 cells/μl. Cell viability and concentration were determined using a LUNA-FX7 Automated Cell Counter (Logos). To ensure a greater sequencing depth per cell using the PacBio platform, our target was to recover ∼700 cells per library preparation. The cells were then combined with a master mix containing reverse transcription reagents. The single-cell 3′ v3.1 gel beads, which carry the Illumina TruSeq Read1, a 16 bp 10x Genomics barcode, a 12 bp UMI, and an oligo-dT primer, were loaded onto the chip along with oil for the emulsion reaction. The Chromium X partitioned the cells into nanolitre-scale gel beads in emulsion (GEMs), where reverse transcription occurred. All cDNAs within a GEM, representing one cell, shared a common barcode. After the reverse transcription reaction, the GEMs were broken, and the full-length cDNAs were captured by MyOne SILANE Dynabeads and then amplified. The amplified cDNA underwent cleanup with SPRI (solid-phase reversible immobilization) beads, followed by qualitative and quantitative analysis using an Agilent 4200 TapeStation High Sensitivity D5000 ScreenTape and Qubit 1X dsDNA High Sensitivity Kit (Thermo Fisher Scientific). The same single-cell full-length cDNA generated using the 10x Genomics Chromium Single Cell 3′ Reagent Kits (v3.1 Chemistry Dual Index) was used to prepare Illumina and PacBio sequencing libraries, as described below.

#### Illumina library preparation and short-read sequencing

The cDNA was enzymatically sheared to a target size of 200–300 bp, and Illumina sequencing libraries were constructed. This process included end repair and A-tailing, adapter ligation, a sample index polymerase chain reaction (PCR), and SPRI bead clean-ups with double-sided size selection. The sample index PCR added a unique dual index for sample multiplexing during sequencing. The final libraries contained P5 and P7 primers used in Illumina bridge amplification. Sequencing was performed using paired-end 28–91 bp sequencing on an Illumina NovaSeq 6000 to achieve ∼300 000 reads per cell.

#### MAS-ISO-seq library preparation and long-read sequencing

The cDNA, with the input amount of 45 ng/sample, was directed for single-cell MAS-ISO-seq libraries preparation using the MAS-ISO-seq for 10x Genomics Single Cell 3′ Kit (Pacific Bioscience, CA, USA). Firstly, TSO priming artefacts generated during 10x Genomics cDNA synthesis were removed in the PCR step with a modified PCR primer (MAS capture primer Fwd) to incorporate a biotin tag into desired cDNA products followed by their capture with streptavidin-coated MAS beads. cDNA free from TSO artefacts was further directed for the incorporation of programmable segmentation adapter sequences in 16 parallel PCR reactions/samples followed by directional assembly of amplified cDNA segments into a linear array. Such formed MAS arrays with the average length of 10–15 kb were further DNA damage repaired and nuclease treated in order to produce final single-cell MAS-ISO-seq libraries, which quantity and quality were measured by Qubit 1X dsDNA High Sensitivity Kit (Thermo Fisher Scientific) and pulse-field capillary electrophoresis system Femto Pulse (Agilent), respectively. Each single-cell MAS-ISO-seq library was used to prepare the sequencing DNA-polymerase complex using 3.2 binding chemistry (Pacific Biosciences) and further sequenced on a single 8M SMRT cell (Pacific Biosciences), on Sequel IIe sequencer (Pacific Biosciences) yielding ∼2M HiFi reads and ∼30M segmented reads/sample.

### Data analysis

#### Short-read sequencing (short-read processing)

For direct comparisons with PacBio data, we generated a CellRanger compatible reference for the human genome version GRCh38.p13 and GENCODE annotation v39 using the files available via SMRTLink v11.1. The raw data were mapped to this reference and filtered with CellRanger v7.2.0 [37]. Bam files were subsampled for cell barcodes starting with *AA* sequence, for handling feasibility (reduction in BAM file size from an average of 14.7 Gb to 746 Mb), and analysed using the GenomicAlignments R package v1.38.0 [38] and Rsamtools R package v2.20.0 [39]. The subsampling and analysis were repeated on barcodes starting with *GC* and *TG* sequences. For those reads where the UB tag (corrected UMI) was missing, we substituted it with the UR tag (UMI tag reported by the sequencer). For the analysis of mapped data, PCR duplicates were filtered manually by choosing hierarchically either the mapped and counted read, any mapped read at random if available or the unmapped read. Intronic reads were excluded from UMI counting. The filtered feature counts were converted to a seurat object and filtered for cells common with PacBio data and for protein-coding genes. Ribosomal and mitochondrial content was calculated as the proportion of counts assigned to ribosomal or mitochondrial genes, respectively. Cells common with PacBio were annotated as ccRCC or non-ccRCC (healthy) according to the annotation done by [36]. Pseudo-bulk was generated by obtaining the sum of counts for each gene across all cells. Expression data were then analysed using the seurat workflow (v5.0.1) [5, 40]: the counts were normalized using log normalization with a scale factor of 10 000 and subsequently scaled. We selected 3000 highly variable features using the vst method. Linear dimensional reduction was used to determine dimensionality of the dataset and top 30 PCs were used for non-linear dimensionality reduction analyses (UMAP/tSNE). Cluster-wise differential expression was performed with SCpubr v2.0.2 [41]. For analysis of the proportion of unspliced reads equivalent to the ones filtered out as intra-priming, RT switching or low coverage/non-canonical isoforms in PacBio data, we used the bam files subsampled down to cell barcodes starting with *AA* sequence and, out of all the subsampled reads, we checked how many reads were compatible with the splicing of the reference transcripts (using GenomicAlignments R package).

#### Long-read sequencing (long-read processing)

Long-read sequencing data were processed using the ‘Read Segmentation and Iso-Seq workflow’ within SMRTLink v11.1. For two samples (Normal and ccRCC_2), for each of which three SMRT cells were sequenced, all the sequencing data were merged before analysis to increase data coverage. No confounding batch effects were observed before merging. Briefly, within the pipeline, HiFi reads (high fidelity CCS reads with QV > 20) were de-concatenated into segmented reads based on segmented adapters with *skera* tool. The reads were then processed with *isoseq* (v3.8.1 within SMRTLink v11.1) for removal of cDNA primers and barcode and UMI tags, reorientation, trimming of polyA tails, cell barcode correction, real cell identification and PCR deduplication via clustering by UMI and cell barcodes. Independently of the SMRTLink pipeline, BLAZE (v2.5.1) was used to confirm real cell identification and find unique barcodes that are likely associated with empty droplets. BLAZE was run with default parameters, including empty droplet detection capped at 2000 barcodes. Reads were then aligned to the GRCh38.p13 genome with *pbmm2 align* (a wrapper around minimap2 [42]). Bam files were analysed using the GenomicAlignments R package, selecting cell barcodes matching the subsampled Illumina cell barcodes [38]. PacBio cell and UMI barcodes are written in reverse complement, and this has been accounted for in our analyses. Unique isoforms were collapsed and classified with *pigeon*, a PacBio provided software based on SQANTI [32, 33]. Data were initially compared to Illumina data without filtering any isoforms. Subsequently, isoforms were filtered following the default parameters of filtering intra-priming, RT-switching and low coverage/non-canonical isoforms. For both datasets a gene count matrix was created with *pigeon make-seurat* with the option ‘--keep-ribo-mito-genes’ for recovery of ribosomal and mitochondrial genes that are by default discarded. Results from both filtering steps were compared for better understanding of the effect of the artefacts on gene counts. Both gene count matrices from PacBio were then subsequently used for comparison with Illumina data subsetted to only protein-coding genes and common cells. Cells were annotated as ccRCC or non-ccRCC (non-malignant) with scGate (v1.6.0) using CA9 as a positive expression marker for ccRCC cells [43]. The annotation was done by Karakulak *et al.* [36]. Ribosomal and mitochondrial content was calculated as the proportion of reads mapped to ribosomal or mitochondrial genes, respectively, using the mapped bam files. Pseudo-bulk was generated by obtaining the sum of counts for each gene across all cells. The data were processed with seurat and SCpubr v2.0.2 following exactly the same workflow as described earlier for Illumina.

#### Comparison

The long- and short-read datasets were compared at the level of (i) transcribed molecules and their alignments and (ii) gene count matrices. Since the protocol generates cDNA molecules tagged with a cell barcode and a UMI, we could directly match and compare the short- and long-reads generated from the same original molecule. We defined for each molecule the tag ID as the combination of cell barcode and UMI and used this tag ID to identify corresponding reads across the technologies. This allowed us to identify molecules preferentially detected by each technology. Additionally, we evaluated characteristics like genomic location of alignments, proportion of artefacts, and proportion of reads discarded from the final counts. The final gene count matrices were subsetted to common cells and protein-coding genes. Each dataset was processed independently (normalized, scaled, and subjected to selection of 3000 highly variable features) before merging and the dimensionality reduction analyses were repeated after merging. Pearson’s correlation coefficient between Illumina and PacBio pseudo-bulk was calculated using stats R package (4.3.2). EdgeR exactTest (from edgeR v4.0.1) with dispersion coefficient of 0.1 was used to select genes with higher counts in Illumina or higher counts in PacBio, including genes missing from either of the datasets. The *P*-values were corrected with the p.adjust function from EdgeR. The length and GC content of each gene were assumed to be the average length and GC content of all the reference isoforms. The dataset was visualized using seurat (v5.0.1) and SCpubr (v2.0.2). Euclidean distance was measured between any two same cells from PacBio and Illumina on a UMAP. The comparison of gene count matrices was done for PacBio data unfiltered of any isoforms and filtered of all isoforms belonging to the category of intra-priming, RT switching or low coverage/non-canonical. The significance of the difference in length distribution of the filtered reads and the unfiltered reads was measured with Kolmogorov–Smirnov test from stats R package (v4.4.0).

## Results

**Figure 1.**
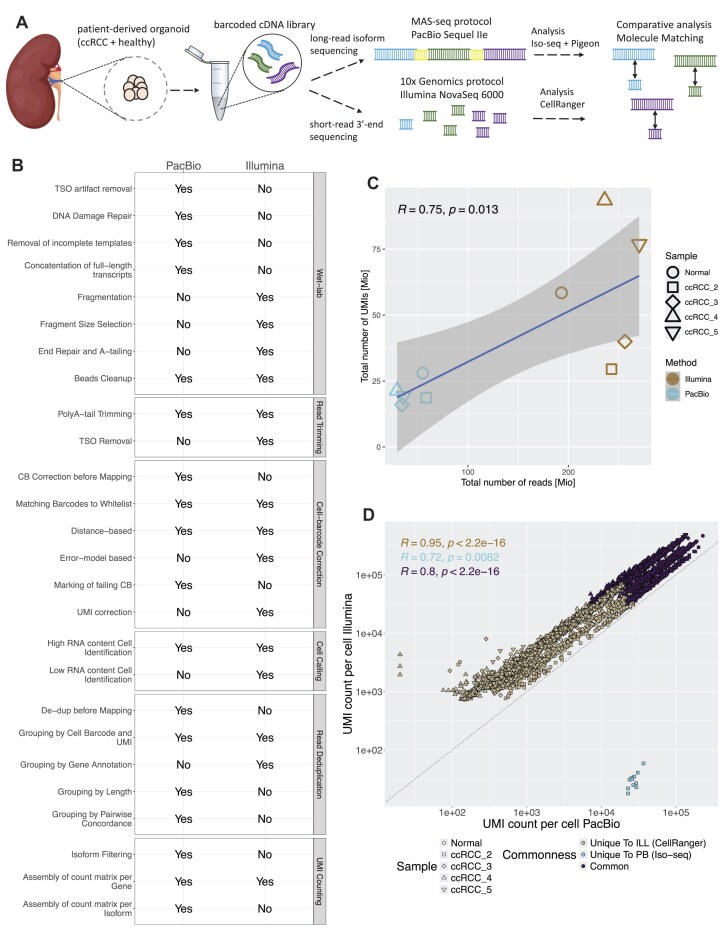
Quality evaluation of long- and short-read sequencing data. (**A**) The scRNA-seq workflow involved four samples of patient-derived ccRCC organoid cells and one sample of healthy kidney organoid cells. The amplified cDNA generated using the 10x Genomics single-cell 3’ gene expression kit was split into two parts. One part was used for library preparation following the 10x Genomics protocol for Illumina sequencing, while the other part was prepared for PacBio sequencing using the MAS-ISO-seq protocol. The two datasets were then processed with state-of-the-art workflows. Long reads were analysed with SMRTLink v11.1 single-cell Iso-Seq pipeline and short reads were processed with CellRanger v7.2.0. (**B**) The differences and similarities between the short-read and long-read datasets in the processing steps of the cDNA libraries (Wet-lab) and in the data analysis steps (including read trimming, cell-barcode correction, cell calling, read deduplication, and UMI counting). *Cell barcode correction for Illumina data in CellRanger is both distance-based, because only cell barcodes with sequence within 1-Hamming-distance away are corrected, and error-model-based, because it explicitly incorporates sequencing error likelihoods via base quality scores to compute a posterior probability a barcode originated from the whitelist to use it for replacement. Cell barcode correction for PacBio data in Iso-Seq is only distance-based because a barcode is replaced with a whitelist barcode with the lowest edit distance and lowest Hamming distance (only if the edit distance is below 2). (**C**) Pearson correlation between the number of reads obtained from both sequencing methods and the total number of UMIs. Here we count PacBio segmented reads. (**D**) Pearson correlation between the number of UMIs per cell for all cells coloured by whether they were identified in both datasets, identified uniquely in Illumina by CellRanger (Unique to ILL) or whether they were uniquely identified in PacBio by Iso-seq (Unique to PB).

We generated short- and long-read sequencing data from four samples of patient-derived organoid cells of ccRCC complimented with a matched sample of healthy kidney organoid cells. For both sequencing methods, we used the same cDNA generated with 10x Genomics single-cell 3′ reagent kit (v3.1 Dual Index) (Fig. 1A). The short-read sequencing library further underwent fragmentation and size selection and was then sequenced on NovaSeq 6000; therefore, we refer to the data as ‘Illumina data’ throughout the manuscript. The count matrix for the Illumina data were generated with CellRanger (Fig. 1A). For the long-read sequencing we subsequently followed the MAS-ISO-seq protocol and sequenced the libraries on Sequel IIe. The count matrix for the PacBio data were generated with the ‘Read Segmentation and Single-Cell Iso-Seq’ workflow in SMRTLink (see the ‘Materials and methods’ section for more detailed description) (Fig. 1A). For making our comparison practical, we applied lab protocols commonly used in the field and used state-of-the-art workflows for the analysis of both datasets and in Fig. 1B we present the major differences between them. The data were purposefully targeted for a low number of cells (∼700) with a deep coverage per cell.

### Number of cells detected from short-read sequencing is higher, but those cells are of lower quality

With PacBio sequencing, we obtained between 29.4 and 58.3 million segmented reads (generated from 1.88 to 3.79 million HiFi reads of the concatenated constructs) with the average number of reads per cell ranging from 21 499 to 96 620 (Table 1). For Illumina data, we obtained between 4.5–6.5× more reads with a total of 193–271 million reads per sample, averaging between 141 887 and 442 530 reads per cell (Table 1). The difference in the number of reads did not translate proportionally into a difference in the number of UMIs found, indicating a high level of redundancy in Illumina data. The total number of UMIs per sample was ∼3× higher in Illumina than in PacBio (1.6–4.4×, Table 1 and Fig. 1C) and the number of UMIs per cell, for the cells shared between the two methods, also increased by an average of 3× in Illumina compared to PacBio (1.2–6.5×, Fig. 1D).

**Table 1.** Summary statistics for short-read Illumina sequencing data and long-read PacBio sequencing data

	Normal		ccRCC_2		ccRCC_3		ccRCC_4		ccRCC_5	
	Illumina	PacBio	Illumina	PacBio	Illumina	PacBio	Illumina	PacBio	Illumina	PacBio
Total reads (Mio)	193.1	54.9	242.9	58.3	256.3	34.2	236.1	29.4	270.4	35.6
Total cells	827	437	549	373	588	310	1664	1091	1155	388
Mean reads per cell	233 514	82 703	442 530	96 620	435 890	70 423	141 887	21 499	234 171	57 006
Reads in cells (%)	72	68.1	73	64.8	77	67.8	86	83.3	82	64.5
Deduplicated reads (Mio)	NA	27.8	NA	18.5	NA	15.8	NA	21.1	NA	19.1
Reads mapped (Mio)	175.5	27.6	222.1	18.5	218.9	15.7	221.7	21	255.1	19
Reads mapped (%)	90.90	99.5	91.40	99.6	85.40	99.4	93.90	99.6	94.30	99.7
Mean genes per cell (Common)	7354	6208	5981	5468	7043	5564	6443	3595	8045	5626
Total UMIs (Mio)	58.4	28	29.5	18.6	40	16	93.5	21.3	76.9	19.2
Mean UMIs per cell	70 651	35 838	52 616	37 282	67 970	29 014	56 192	13 433	66 561	19 083

**Figure 2.**
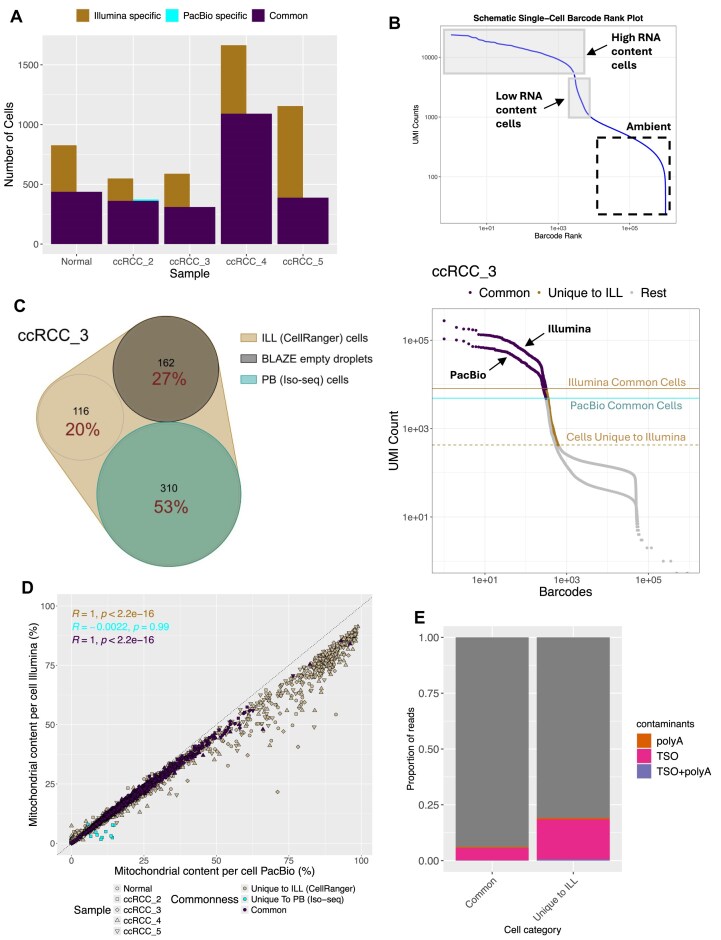
Quality evaluation of long- and short-read sequencing data. (**A**) Number of unique and common cells per sample. (**B**) A schematic cell barcode rank plot showing the distinction between the high RNA content and low RNA content cells coupled with a cell barcode rank plot for Illumina and PacBio data from sample ccRCC_3 showing cell barcodes colour-coded by whether they were identified as real cells in both PacBio and Illumina datasets (Common) or uniquely in Illumina (Unique to ILL, the low RNA content cells). The vertical lines aid in visual cutoffs and show the difference in the number of UMIs per cell for the cells common between PacBio and Illumina. (**C**) Venn diagram showing the sharing of cell barcodes identified as real cells by CellRanger in Illumina data (ILL) with cell barcodes identified as real cells by Iso-seq in PacBio (PB) data and barcodes identified as empty droplets by BLAZE in PacBio data (cell barcodes with counts below an estimated quantile-based threshold and with an edit distance of 5 from the cell-associated barcodes) for sample ccRCC_3. Percentage labels indicate the percentage of Illumina cell-associated barcodes. (**D**) Mitochondrial content per cell for all cells is coloured by whether they were Common (the high RNA content cells identified in both datasets), Unique to ILL (cells identified uniquely in Illumina by CellRanger), or Unique to PB (uniquely identified in PacBio dataset by Iso-seq). (**E**) The proportion of TSO and polyA contamination in reads unique to Illumina and Illumina reads shared with PacBio across all samples.

Although different numbers of cells were called by the short- versus long-read data processing algorithms, a common set of cell barcodes were identified in both datasets (Fig. 2A). All cells detected by Iso-Seq3 were also called by CellRanger, except for 12 cells unique to PacBio from sample ccRCC_2, for which there were fewer than 60 UMIs per cell in Illumina (Figs 1D and 2A). CellRanger detected additional cells in Illumina for all samples, which stemmed from the difference in the cell calling algorithm. Iso-Seq3 by default uses a knee finding method to determine real cells (high RNA content cells), which takes into account the number of UMIs per cell (Fig. 2B and Supplementary Fig. S1) (Iso-Seq docs, Cell Calling Documentation, https://isoseq.how/umi/cell-calling.html, accessed December 2024). CellRanger additionally uses the RNA profile and retains those barcodes with UMI counts below the threshold but whose RNA profile strongly disagrees with the background model (low RNA content cells) (Fig. 2B and Supplementary Fig. S1) (Cell Ranger’s Web Summary Barcode Rank Plot, https://www.10xgenomics.com/support/software/cell-ranger/latest/advanced/cr-barcode-rank-plot, accessed December 2024). The method is sequencing depth-dependent and so, with the unusually high depth per cell in this study, led to a high discrepancy in the number of called cells between the two methods. Indeed, for the CellRanger-specific cell, we observed a low number of UMIs per cell barcode in both Illumina and PacBio (Fig. 1D). The cells identified in both methods captured 92.2% of the UMIs in Illumina data while the CellRanger-specific cells captured only 7.8%. For a fairer comparison, we used BLAZE for empty droplet detection in PacBio data. Within the second step of the pipeline, BLAZE classifies unique barcodes with counts above a quantile-based threshold as cell-associated barcodes. Out of the rest, BLAZE picks out unique barcodes with an edit distance of 5 from the cell-associated barcodes and classifies them as empty droplets (BLAZE Release Notes v2.5.1, https://github.com/shimlab/BLAZE/blob/v2.5.1/README.md, accessed 15 December 2024) [30]. We provided the number of cells identified by CellRanger from Illumina data as the expected number of cells, which was used by BLAZE for calculation of this quantile-based threshold for determining the number of cell-associated barcodes. We still found that 9%–32% of these CellRanger-specific cells were classified by BLAZE as empty droplets in the PacBio dataset (Fig. 2C and Supplementary Fig. S2).

We evaluated the quality of all cells by looking at the number of genes mapping to mitochondrial genes and at the proportion of contamination by sequencing artefacts. High mitochondrial content can be an indication of apoptotic, lysing, low-quality cells [44]. The proportion of reads mapping to mitochondrial genes linearly correlated between Illumina and PacBio data from the shared cells (*R* = 1, *P*< .05) but mitochondrial content was elevated in cells detected uniquely in Illumina data (16.2% versus 25.6%) (Fig. 2D). Approximately 31.2% of Illumina-specific cells had mitochondrial content of above 30% in contrast to 6.8% of the cells shared with PacBio (Fig. 2D). It must be noted that the mitochondrial genes are completely filtered out of the PacBio data by the SMRTLink single-cell Iso-Seq pipeline (v11.1), so filtering cells based on the mitochondrial DNA content in the count matrix is not possible, the data must be recovered manually.

The quality of the cDNA can also be assessed using the proportion of Illumina short reads containing TSO or stretches of adenine in the sequence. As part of the 10x Genomics 3′ cDNA preparation, polyadenylated (polyA) RNA is reverse transcribed into cDNA using anchored oligo dT, in the presence of the TSO [45]. An increased presence of TSO-containing constructs can indicate RNA degradation or significantly shorter RNA than expected prior to the reverse transcription reaction. Cells undergoing a normal apoptotic pathway can also have shorter transcripts undergoing degradation, which results in TSO hybridising close to the polyA tail (10x Genomics, Why do a fraction of my Visium reads contain the TSO at the beginning of Read 2? https://kb.10xgenomics.com/hc/en-us/articles/360041690731-Why-do-a-fraction-of-my-Visium-reads-contain-the-Template-Switch-Oligo-TSO-at-the-beginning-of-Read-2, accessed July 2024). TSO artefacts can be also created by mispriming, internal priming or incomplete template switching. The MAS-ISO-seq protocol has a step of TSO-artefact removal (PacBio Application note—MAS-Seq for single-cell isoform sequencing, https://www.pacb.com/wp-content/uploads/Application-note-MAS-Seq-for-single-cell-isoform-sequencing.pdf, accessed March 2024). We observed that the proportion of TSO artefacts in Illumina data from cells shared with PacBio was lower (6.4%) than in cells unique to Illumina (19.6%) (Fig. 2E), which contributed to the lower mapping rate of the Illumina data. Around 99.6% of PacBio reads mapped to the GRCh38.p13 human genome in contrast to an average of 91.2% of the Illumina reads (with 81.6% of the reads mapped confidently with a MAPQ of 255).

### Short-read sequencing provides more data but consists of a higher proportion of multi-mapping reads

**Figure 3.**
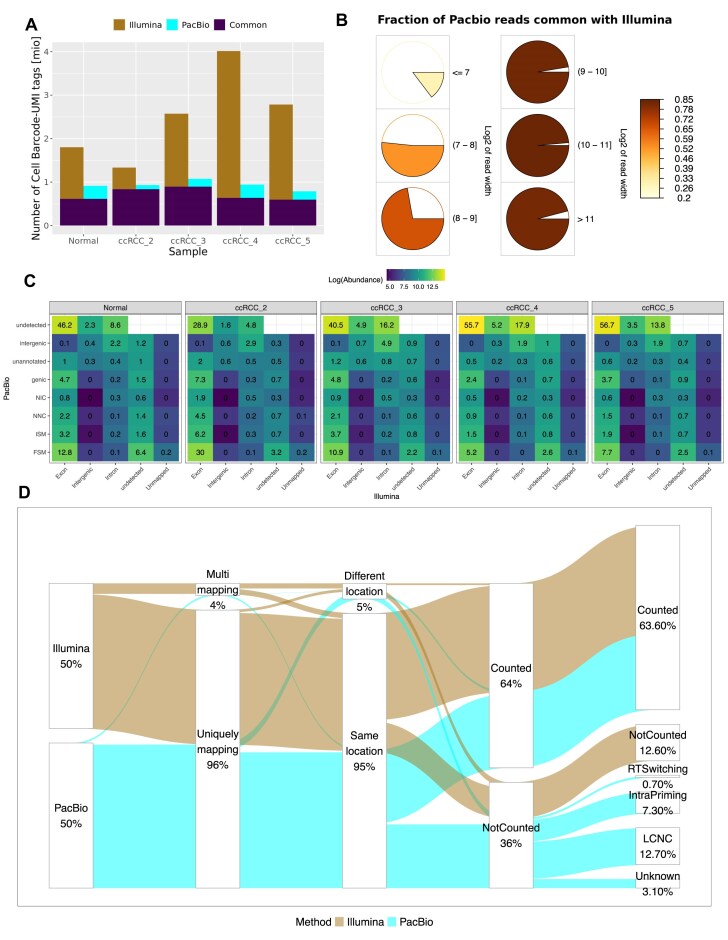
Mapping summary for short-read and long-read sequencing data. (**A**) Number of common and unique combinations of cell barcode + UMI considered in the comparison of bam files (mapped data) between PacBio and Illumina. (**B**) Fractions of PacBio reads shared with Illumina represented across different real-length categories (log_2_ of read length, 7–11). (**C**) Logged abundance of cell barcode–UMI tags across different types of PacBio-specific and Illumina-specific annotations. The unannotated reads from PacBio are reads discarded due to mapping chimerically or mapping with poor identity. Illumina additionally has a set of reads that were unmapped (but were mapped by PacBio). The labels indicate the percentage of all tag IDs. (**D**) Sankey plot visualising the proportion of tag IDs shared between Illumina and PacBio that are uniquely mapping or multi-mapping, that map to the same location in the genome or to a different location, that are counted or not into the gene count matrix and the types of PacBio-specific artefacts they belong to when not counted (discarded as artefacts).

Since the same cDNA has been sequenced with both methods, we could compare the Illumina and PacBio readouts for the same original cDNA molecules, with the same cell barcodes and UMIs. For these per-molecule comparisons, we subsampled the mapped data to ∼5% of the cells for handling feasibility, and we repeated the subsampling three times for comparison (Supplementary Fig. S3). For Illumina data the number of unique cell barcode-UMI combinations (tag IDs) ranged from 1.3 million to 4 million with an average of 106 876 UMIs per cell (Fig. 3A). PCR deduplication occurs only after mapping, clustering the reads by UMI and cell barcodes and taking the read coordinates into account. Thus, for each cell barcode−UMI tag, there were on average 2–6 reads. For PacBio data, the number of unique tag IDs ranged from 0.79 million to 1.07 million with an average of 39 762 UMIs per cell (Fig. 3A). PacBio reads undergo PCR deduplication via clustering by UMI and cell barcodes prior to mapping. Deduplication reduced the number of reads per sample by 50% to between 15.9 and 27.8 million reads. Therefore, the number of reads per unique tag ID in the bam files ranged only from 1 to 1.02. We deduplicated the Illumina data by selecting hierarchically either the counted read for each tag ID if available, one read at random from all mapped reads or the unmapped read. For PacBio data, we selected one read for all the tag IDs, preferentially selecting the longest read.

Short-read sequencing clearly provided a higher coverage. The common tag IDs averaged to 715 769, leaving only on average 23.3% of tag IDs unique to PacBio but as much as 66.2% unique to Illumina (Fig. 3A).

However, we found ∼0.75% of the common tag IDs to be mapped in PacBio data but entirely unmapped in Illumina data and an additional 3.7% of tag IDs to have at least one unmapped read in the Illumina dataset out of the multiple reads per tag ID. The average length of those PacBio reads with an unmapped equivalent in the Illumina data was shorter than the average length of reads common between Illumina and PacBio (608 versus 798 bp) but the same was observed for all the tag IDs uniquely identified by PacBio data and missing from Illumina data (618 versus 798 bp) (Fig. 3B). Indeed, PacBio tag IDs that were also found in Illumina showed an underrepresentation of reads shorter than 500 bp (Fig. 3B). The reads undetected by Illumina represented valuable information—on average 53% mapped to isoforms labelled as fully overlapping with reference transcripts (full splice match, FSM), or matching consecutive but not all splice junctions of the reference transcripts (incomplete splice match, ISM) [33] (Fig. 3C). The two isoform categories were represented in the same proportions in the reads common with Illumina (52%) (Fig. 3C).

Due to isoform resolution, the PacBio workflow provides a much more detailed classification of the detected transcripts. In contrast to the exonic, intronic and intergenic read classification inferred from short-read data, SQANTI, on which the PacBio single-cell Iso-Seq pipeline is based, classifies the transcripts according to the splice junctions, donor and acceptor sites and distinguishes between the previously mentioned FSM and ISM transcripts but also between two novel isoform categories, including novel in catalog (NIC, isoforms presenting combinations of known splice donors and acceptors) and novel not in catalog (NNC, isoforms harbouring at least one unknown splice site) [32, 33]. The rarer categories include fusion or intergenic isoforms, as well as genic isoforms containing both introns and exons. We found a large overlap between the annotations provided by the two methods; most of the common tag IDs were annotated as exonic by Illumina and as FSM by PacBio and the majority of reads annotated as intergenic or intronic by Illumina were annotated as intergenic or were discarded before annotation by PacBio (reads that mapped with low identity or chimerically) (Fig. 3C). However, not all annotations agreed. A large proportion of Illumina exonic tag IDs were annotated as genic (unspliced) by PacBio, or tag IDs annotated as novel isoforms by PacBio were only detected as intronic or intergenic in Illumina, with the annotation limited by the read length (Fig. 3C). The tag IDs uniquely detected by one technology in majority mapped to exons (Illumina) or as previously mentioned, were categorized as FSM (PacBio), clearly indicating that both technologies capture useful information missing from the other dataset (Fig. 3C).

The reads with common tag IDs found in both datasets were not equally counted to the gene matrix. Illumina retained a higher proportion of tag IDs (75% versus 52%), but PacBio discarded those tag IDs as artefactual for reasons that can’t be inferred from short-read data due to lack of full transcript resolution (Fig. 3D). Majority of the reads were discarded for mapping to intrapriming isoforms (with accidental priming of A’s within transcript sequence [23]) or to low coverage isoforms with non-canonical splice junctions (LCNC, defined as isoforms with the coverage below 3 for splice sites that are not in the known ‘canonical’ set of splice sites, [Iso-seq Docs, Pigeon Output, https://isoseq.how/classification/pigeon-output.html, accessed March 2024)]. Illumina data consisted of a higher proportion of multi-mapping tag IDs (7.2% versus 1.1%) with most (∼67%) discarded from the gene count matrix (Fig. 3D). All of the multi-mapping reads in PacBio were eliminated from final counts. Of the multi-mapping Illumina tag IDs, 48% mapped to a different genomic location than the PacBio reads, and 93% of those were not counted into the gene count matrix. In comparison, only 2% of the uniquely mapping tag IDs mapped to a different location than the PacBio equivalent reads (Fig. 3D).

### Gene expression results are highly comparable between long-read and short-read sequencing data

Next, we evaluated the gene count matrix constructed from both datasets. For Illumina data, before the UMI count, CellRanger corrects UMI and cell barcode sequences and removes PCR duplicates. In our analysis, we counted only reads mapping to exons. For PacBio data, *pigeon* was used to generate the gene-level count matrix. At this step we did not apply any isoform filtering.

**Figure 4.**
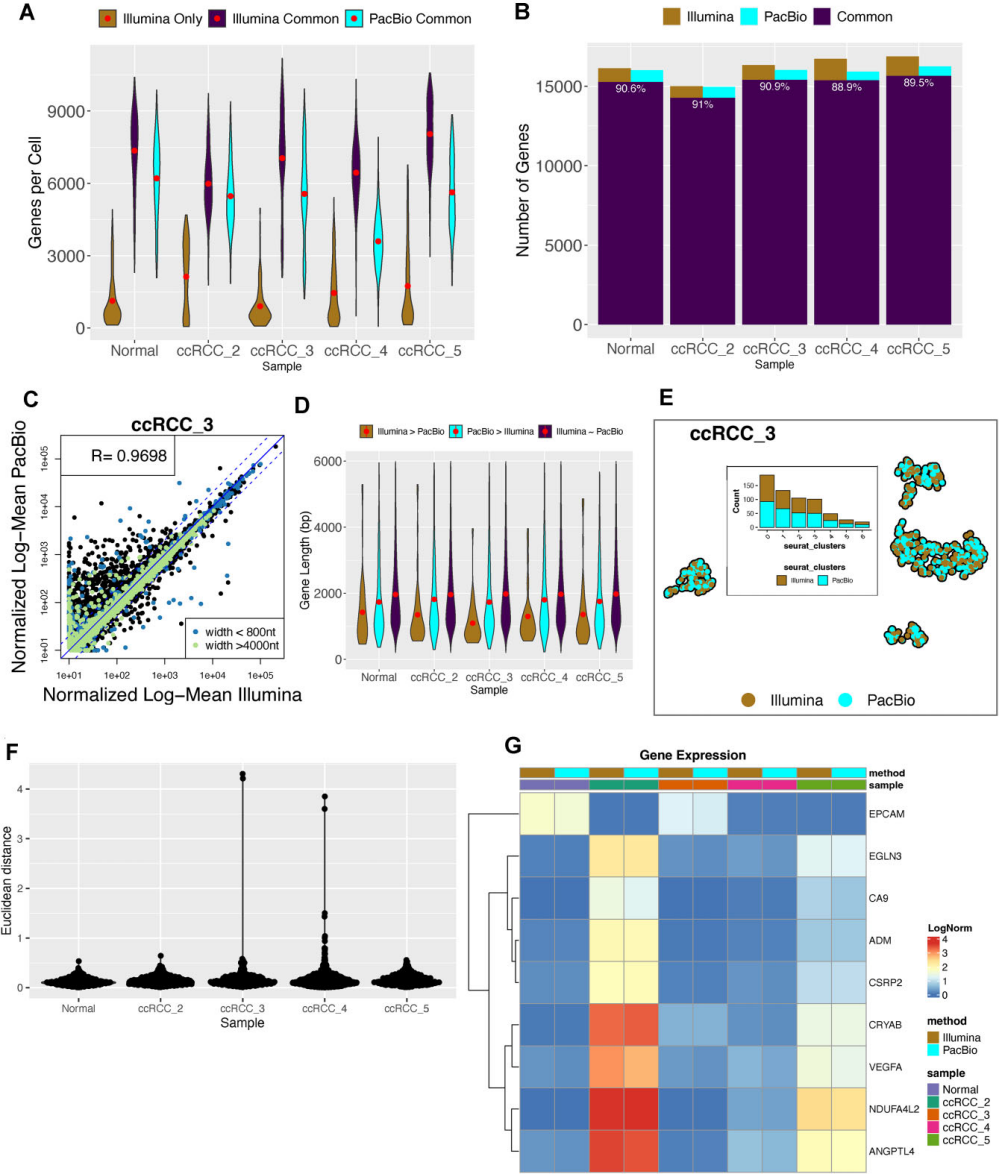
Comparison of gene count matrices between short-read and long-read sequencing data unfiltered of artefacts. (**A**) The distribution of genes per cell for cells unique to Illumina, for Illumina cells common with PacBio and PacBio cells. The red dot indicates the mean. (**B**) The number of genes unique and common between PacBio and Illumina from the set of commonly identified cells. The printed number indicates the proportion of all genes detected by both methods that were common. (**C**) Pearson correlation between PacBio (unfiltered of any isoforms) and Illumina data in the log-mean normalized sum of counts per gene across all common cells coloured by gene length in ccRCC_3 sample. (**D**) Gene length of genes with significantly higher counts in Illumina (edgeR exactTest, log fold change (logFC) > 1, False Discovery Rate (FDR) < 0.05), with counts equal between Illumina and PacBio and with significantly higher counts in PacBio (edgeR exactTest, logFC < 1, FDR < 0.05). The red dot indicates the mean. (**E**) UMAP embeddings for sample ccRCC_3, coloured by sequencing method. Embedded: bar plot representing the number of cells from each method in each cluster. (**F**) Euclidean distance between cells with the same barcodes from Illumina and PacBio (unfiltered) on the UMAP plot shown in panel (E). (**G**) Log normalized gene expression of marker genes for ccRCC and epithelial cells split by sample and data type.

As previously mentioned, Illumina data and workflow called more cells, but we observed those cells to be of much lower quality than the cells found in both datasets. The mean number of genes per cell in cells common to Illumina and PacBio was above 5981 across all samples, whereas for those cells unique to Illumina, the mean number of genes did not exceed 2127 across all samples (Fig. 4A). The mean number of genes in PacBio data varied between 3595 and 6208 (Fig. 4A). We thus limited the data only to cells called by both methods.

Approximately 90% of all the detected genes were shared by both methods (Fig. 4B). In Illumina data, we detected an average of 16 212 protein-coding genes associated with an average of 30.3 million total UMIs, whereas in long-read data we detected 15 832 genes associated with an average of 11.9 million UMIs (Fig. 4B). Normalized and scaled sum of counts across all cells (pseudo-bulk) strongly correlated between the two methods (0.92 < *R* < 0.97, *P-value* < .05, Fig. 4C and Supplementary Fig. S4) but we observed 10× more genes to have significantly higher counts in PacBio (a mean of 577 genes versus 53 genes, logFC > 1, FDR < 0.05) (Supplementary Fig. S4). Majority of genes, which were not significantly divergent in counts between the two methods, had higher counts in Illumina, which explains the distribution of the generally higher UMI count in Illumina. The average length of genes with counts higher in PacBio (1905 bp) was in the same range as the average length of genes with concordant counts between the two methods (2080 bp) (Fig. 4D). However, the average length of genes with higher counts in Illumina (1423 bp) was significantly lower (*t*-test, *P*-value <.05, Supplementary Fig. S5) (Fig. 4D). The average GC content for genes with higher counts in Illumina was 56% with 40% having GC content greater than 60% whilst for the other two groups the average GC content did not exceed 52% (Supplementary Fig. S6). The results are consistent with GC content bias known to occur in Illumina sequencing data [46].

To understand whether the genes with incongruent counts had a significant impact on the overall gene expression results, we merged the two datasets, projected the gene expression onto two-dimensional embeddings using UMAP and measured the Euclidean distance between any two same cells. We found the datasets to closely overlap (Fig. 4E and Supplementary Fig. S7), with an equal number of cells in each cluster and with the Euclidean distance being lower than 1 for all but 2 to 5 cells for two samples (Fig. 4F).

The cell types were annotated according to CA9 expression as ccRCC and non-ccRCC (not malignant) cells, following the annotation in [36]. In ccRCC_2, ccRCC_4, and ccRCC_5, we detected 96.8%, 3.8%, and 56% of cells expressing CA9, respectively. We looked at the expression of the CA9 gene as well as other markers characteristic of ccRCC [47–51] and epithelial cells [52], expected to be present at high abundance in non-ccRCC cells, and found no difference in relative gene expression between PacBio data and Illumina data (Fig. 4G).

### Isoform resolution provides more information on the sequencing artefacts

PacBio distinguishes three types of artefactual transcripts: intra-priming (with accidental priming of adenine stretches in intronic or exonic regions of a transcript), RT-switching (reverse transcriptase template switching), and reads matching to low coverage isoforms with non-canonical splice junctions (LCNC) [32, 33]. To investigate the impact of those different artefacts on the results, we evaluated the gene count matrix generated after isoform filtering and compared it to the counts obtained from Illumina data.

**Figure 5.**
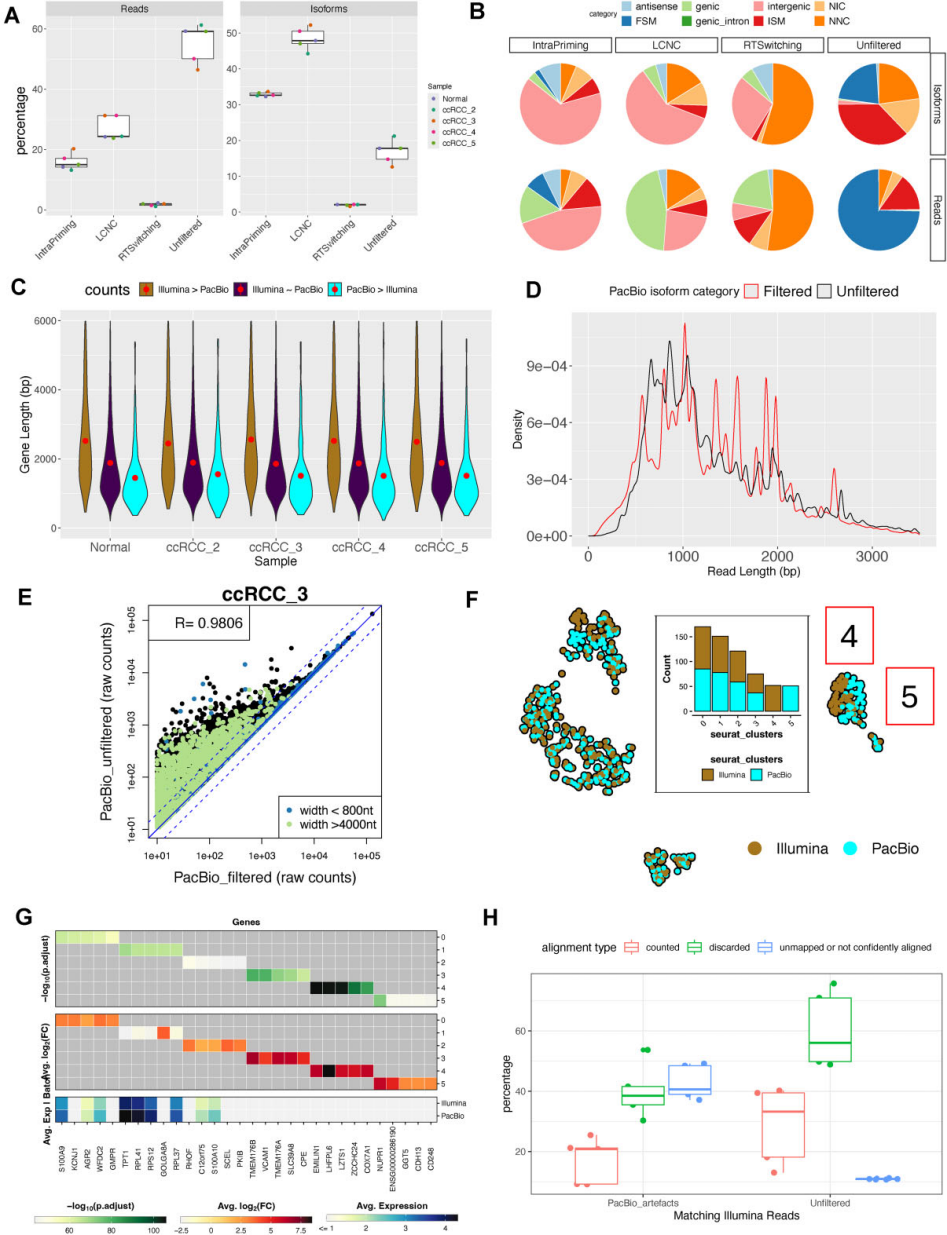
Comparison of gene count matrices between short-read data and long-read data filtered of artefacts. (**A**) Proportion of reads and isoforms in PacBio data filtered out due to mapping to intra-priming, low coverage/non-canonical (LCNC) and RT switching isoforms and the reads remaining (unfiltered). (**B**) Sub-categories of artefactual and remaining reads and isoforms according to the sub-categories assigned by *pigeon* by sample. (**C**) Gene length of genes with significantly higher counts in Illumina (edgeR exactTest, logFC > 1, FDR < 0.05), with counts equal between Illumina and PacBio and with significantly higher counts in PacBio (edgeR exactTest, logFC < −1, FDR < 0.05). The red dot indicates the mean. (**D**) Read length distribution of unfiltered reads (black) and reads mapping to artefactual isoforms (red). (**E**) Pearson correlation between PacBio filtered isoforms and PacBio unfiltered data in the sum of counts per gene across all cells coloured by gene length category across all samples. (**F**) UMAP embeddings for sample ccRCC_3, coloured by sequencing method. Here, PacBio data is filtered of all artefactual isoforms. Clusters 4 and 5 are labelled on the UMAP and the embedded plot shows the number of cells in each cluster. (**G**) Differentially expressed genes per cluster–clusters 4 and 5 are the two adjacent clusters of same cells from Illumina and PacBio, respectively. (**H**) Alignment type of Illumina reads equivalent (with the same cell barcode–UMI tag IDs) to PacBio artefacts and to unfiltered reads. The data is subsampled to the same cells analysed in the bam file comparison. ‘Counted’ indicates that the read has been counted to the final gene count matrix.

Across all our samples an average of 45% of reads and 82% of isoforms were flagged as belonging to any of these categories of artefacts and thus discarded from the final counts (Figs 3D and 5A, and Table 2). Majority of the artefactual reads (27%) and isoforms (48%) were classified as the low coverage/non-canonical (Fig. 5A) with over half of them being genic (containing both exons and introns) or intergenic and none of them being classified as FSM (Fig. 5B). Removal of all artefacts led to a decrease in UMI counts to an average of 9.3 million (77% of the unfiltered data) and a decrease in the number of detected genes to 14 334 (to 90% of the genes detected in unfiltered data). The number of genes with counts higher in Illumina pseudo-bulk significantly increased (from 53 to 1549), weakening the gene expression correlation between the two methods (0.9 < *R* < 0.95). The set of genes with counts higher in Illumina were on average longer (2775 bp) than genes with equal counts from the two methods (1950 bp) (Fig. 5C). The average length of reads with counts higher in PacBio decreased to between 1630 bp indicating a bias towards higher counts for shorter genes (Fig. 5C). Upon investigation of the length of the filtered PacBio transcripts, we observed a bias towards longer length across all samples (Kolmogorov–Smirnov, 0.10 < KS < 0.11, *P*-value <.05) (Fig. 5D). We compared the non-normalized pseudo-bulk gene expression between counts from unfiltered PacBio data and PacBio data filtered of artefacts and we observed a considerable decrease in counts for genes longer than 4000 bp (Fig. 5E and Supplementary Fig. S8). Genes longer than 4000 bp represented 7% of the total expressed genes but in the set of genes experiencing a significant decrease in counts (edgeR exactTest, logFC > 1, *P*-value <.05) they represented 18%.

**Table 2.** Types of SQANTI3 categories identified in filtered and unfiltered PacBio data

	Normal		ccRCC_2		ccRCC_3		ccRCC_4		ccRCC_5	
Number of isoforms	Unfiltered	Filtered	Unfiltered	Filtered	Unfiltered	Filtered	Unfiltered	Filtered	Unfiltered	Filtered
FSM	83 112	71 205	80 013	68 352	60 413	50 983	73 925	62 582	75 223	64 378
ISM	211 749	126 715	167 345	101 665	182 925	96 380	192 830	109 263	176 458	102 536
NNC	313 881	83 308	271 450	73 820	162 679	37 997	248 839	62 710	248 491	72 372
NIC	179 820	52 740	147 796	49 142	138 756	23 054	175 605	42 698	162 261	49 049
**Number of reads (mio)**	**Unfiltered**	**Filtered**	**Unfiltered**	**Filtered**	**Unfiltered**	**Filtered**	**Unfiltered**	**Filtered**	**Unfiltered**	**Filtered**
FSM	11.6	11.3	8.3	8.1	5	4.9	7.3	7.1	7.9	7.7
ISM	3.4	2.4	1.9	1.4	1.9	1.1	2.2	1.5	2.2	1.5
NNC	2.4	0.8	1.4	0.5	1.1	0.35	1.6	0.49	1.8	0.77
NIC	1.1	0.6	0.76	0.42	0.68	0.24	1	0.46	0.96	0.51
**Number of artefactual isoforms:**	**Unfiltered**	**Filtered**	**Unfiltered**	**Filtered**	**Unfiltered**	**Filtered**	**Unfiltered**	**Filtered**	**Unfiltered**	**Filtered**
IntraPriming	627 046	0	464 971	0	578 805	0	640 703	0	562 873	0
RTSwitching	40 830	0	29 723	0	27 274	0	41 626	0	32 583	0
LC/NC	930 901	0	632 512	0	898 466	0	991 402	0	795 851	0
**Number of artefactual reads (mio)**	**Unfiltered**	**Filtered**	**Unfiltered**	**Filtered**	**Unfiltered**	**Filtered**	**Unfiltered**	**Filtered**	**Unfiltered**	**Filtered**
IntraPriming	3.6	0	2.3	0	2.8	0	3.3	0	2.7	0
RTSwitching	0.59	0	0.2	0	0.28	0	0.28	0	0.36	0
LC/NC	6.2	0	4.2	0	4.5	0	6.1	0	4.3	0

We again merged the filtered PacBio data with the Illumina data and projected the gene expression of the two datasets onto a UMAP (Fig. 5F and Supplementary Fig. S9). With the decrease in the number of genes and UMI counts per cell, for 56% of cells, we observed an increase in the Euclidean distance between the same cells in PacBio and Illumina data of up to 50× the distance measured in the clustering analysis of unfiltered PacBio data with Illumina. However, for ∼15% of cells per sample, we observed an improvement in clustering with a decrease of Euclidean distance by 1%–50% of the distance measured from the unfiltered PacBio data. The number of cells with Euclidean distance greater than 1 ranged from 1 to 44 cells, the latter being from the ccRCC_3 sample where the same cells from Illumina and PacBio formed two adjacent but distinct clusters (Fig. 5F). The differential clustering was driven by genes such as ZCCHC24, EMILIN1, LZTS1, or LHFPL6 for which 60% of isoforms from PacBio were filtered out due to being flagged as low coverage/non-canonical (Fig. 5G).

To review whether removal of those reads leads to over filtering, we looked at the quality of the equivalent reads (selecting by tag IDs) in the subsampled Illumina data analysed earlier. We split the reads into those with tag IDs filtered or retained in the PacBio workflow. We found the set of reads matching the filtered PacBio reads to consist of a higher proportion of unmapped reads (43% versus 11% on average) and a lower proportion of reads confidently aligned and counted (17% versus 29% on average) (Fig. 5H). Additionally, we found a higher proportion of those reads to be annotated as not matching the reference transcript, so as unspliced (41% versus 13%). The proportion of unspliced reads corresponded to the proportion of genic (containing both exons and introns) reads identified in the filtered PacBio data.

## Discussion

Long-read sequencing applied to scRNA-seq allows for deciphering of cell heterogeneity based on full-length transcripts providing isoform and variant information. Recent developments have expanded the use and interest in long reads in the field [21, 53] and allowed their decoupling from short reads. However, evidence suggests variability in the information obtained by both methods [23]. Dondi *et al.* [23] compared the expressed genes captured by both methods and the impact on cell type identification in ovarian cancer cells, but their research did not address the sources of the observed differences between short and long reads. Here, we used single cells from four samples of ccRCC-derived organoids and one matched healthy kidney organoid sample to answer the question of the extent of comparability between the long- and short-read data and to elaborate on the reasons for the observed differences. Using both Illumina short-read and PacBio long-read technologies, we sequenced the same 10x Genomics 3′ cDNA, with each molecule tagged with a cell barcode and UMI, to perform a molecule-by-molecule matched comparison. We focused on the information obtained after mapping of reads to the reference genome and gene-wise UMI counting.

In comparison to the long-read sequencing performed on Sequel IIe and following the company guidelines for the sequencing depth, short-read sequencing provided higher coverage and more UMIs per cell. Despite its lower sequencing depth, long-read sequencing data also consisted of unique transcripts not found in the short-read data. Paradoxically, these were short transcripts of less than 500 bp that are otherwise discarded in Illumina 10x Genomics library protocol due to transcript fragmentation and size-selection steps. Lower depth was not the only reason why transcripts were not recovered in PacBio data—the MAS-ISO-seq library protocol contains a TSO artefact removal step, which removes contaminant constructs with TSO on both ends of cDNA molecules. Short-read sequencing resulted in a higher level of redundancy with more reads per cell barcode–UMI tag ID ending up discarded.

The current state-of-the-art workflows applied to the long- and short-read data differed by the cell calling algorithms. Both of them applied a UMI count threshold for identification of high RNA content cells but CellRanger additionally applied an empty droplet analysis for identification of low RNA content cells in Illumina data (Cell Ranger’s Web Summary Barcode Rank Plot, https://www.10xgenomics.com/support/software/cell-ranger/latest/advanced/cr-barcode-rank-plot, accessed December 2024), which captured, on average 7.8% of the total UMIs. Empty droplet analysis of the long-read data still classified 9%–32% of those cells as ambient RNA. Distinguishing between cells with low RNA content, either due to variable capture and amplification efficiencies across droplets or due to low transcriptional activity, from damaged, apoptotic cells or from empty droplets has posed significant challenges in scRNA-seq research [37, 54–56]. Various methods have been proposed, including filtering of cell barcodes by deviation from the ambient RNA pool (EmptyDrops, [57]) or by characteristics such as nuclear fraction (the proportion of RNA originating from unspliced pre-mRNA) and mitochondrial content (DropletQC, [58]). In our dataset, the low RNA content cells exhibited elevated mitochondrial gene content and contamination with TSO/PolyA artefacts, therefore, we focused our comparison on high RNA content cells. Additionally, we did not specifically target any cell types known for low transcriptional activity such as neutrophils [59]. However, we do recommend including empty droplet analysis in long-read sequencing data analysis for targeting low RNA content cells and evaluating the data on a sample-by-sample basis, especially if the aim is targeting rare cell populations.

As expected, isoform-level resolution and longer read length introduces changes in the data processing and analysis. First, it allowed for mapping of reads otherwise unmapped in short-read data, although the proportion of those reads in total was quite low (below 1%). Second, long-read information allowed for more precise allocation of reads to unique genomic regions. Short-read data had a higher proportion of multi-mapping reads with imprecise or low-confidence mapping locations, discarded from the gene count matrix. Third, isoform information from long reads provided more information on the types of sequencing artefacts and thus lead to more precise filtering of reads for gene expression profiling. We identified on average 35% of isoforms and 18% of reads to be discarded due to being flagged as intra-priming or reverse-transcriptase switching.

It is, however, unclear whether filtering of low coverage/non-canonical isoforms is not carried out too stringently within the pipeline. An average of 48% of isoforms and 27% of reads were identified as belonging to that category. We observed the filtering to have the biggest impact on the UMI counting and to be biased towards transcripts of longer length resulting in decreasing in counts especially for genes longer than 4000 bp. As a result of scale-based normalization we observed an increase in pseudo-counts for genes shorter than 800 bp when compared to short-read data. The higher counts for short genes in PacBio data when compared to Illumina data were also observed by Dondi *et al.* [23], so the result is not specific to our study. The largest proportion of those reads was identified as unspliced (containing introns and exons), also in the short-read data, and approximately a third was identified as novel or as incomplete splice matches. Unspliced transcripts are used for RNA velocity analyses [60] and, as revealed by the Long-read RNA-seq Genome Annotation Assessment Project (LRGASP) Consortium, identification and quantification of lowly expressed and complex transcripts continue to be a challenge in long reads [21], so filtering them out might be leading to loss of valuable information. Our investigation of equivalent Illumina reads did show a higher proportion of low-quality reads, but a lot of the reads were still counted into the final gene count matrix. Experimental validation of those transcripts using RT-PCR [61–63] or RNA-FISH [64] would provide information on whether they are biologically significant or true artefacts. Non-canonical transcripts have been shown to encode tumour-specific antigens; therefore, it would be beneficial to verify the usefulness of this filtering step in the workflow and whether the filtering needs to be done in a sample-specific manner and adjusted for tissue types or conditions [65, 66]. For greater in-depth analysis of the significance and frequency of novel isoforms in ccRCC from the same set of samples, see [36].

Additionally, the same filtering is applied in the pipeline for gene- and isoform-level quantification and further analysis would need to be done to assess whether that should be the case. For instance, intra-priming isoforms can represent valuable information about the gene-level expression while only providing misleading information about the alternative splicing. In order to enhance gene-level quantification filtering of isoforms and associated reads could be bolstered by an addition of orthogonal data, such as PolyAsite, refTSS, or CAGE data, e.g. incorporating filtering by the distance to known genes’ transcription start sites/transcription termination sites [67] or by implementing a machine learning filtering approach available through SQANTI3 [32]. If the aim is to maximize the concordance with short-read sequencing data, our study suggests retaining all reads and isoforms.

Despite the earlier-mentioned differences between short- and long-read data, we observed a considerable overlap in the information provided by both datasets. We found the same set of high RNA content cells that captured most of the reads; we found a high proportion of cell barcode–UMI combinations recovered by both methods. Of all expressed genes, 90% were shared between the two methods and we observed deviation in pseudo-bulk counts for <5% of the genes. Gene expression results per cell connect two same cells in non-linear dimensionality reduction analyses even after isoform filtering and cell markers specific to ccRCC show comparable normalized expression. The choice of either of the sequencing methods lies, therefore, in the research question addressed and in the available funding. The cost of PacBio long-read sequencing remains significantly higher than Illumina short-read sequencing, especially when considering the sequencing throughput, and thus remains less accessible. However, for isoform-resolved expression analysis and for highly complex single-cell samples e.g. with seemingly homogenous gene expression but highly variable phenotypes, the use of long-read sequencing can provide invaluable information.

Our aim was to test the most frequently applied workflows on both datasets, and we do acknowledge the limitations, for example in comparing bam files resulting from two different mapping algorithms. We also sequence a lower number of cells than is perhaps of interest in most scRNA-seq experiments. However, we think that our benchmark analysis identified multiple sources of variations between long- and short-read sequencing technologies that could be mitigated with higher sequencing depth or deviation from the standardized pipelines. The finding is informative and has a general applicability. With increasing throughput in long-read sequencing provided by the Revio system, we would expect even a greater overlap in the obtained data and thus we think long reads provide an alternative to short-read sequencing for scRNA-seq experiments.

## Supplementary Material

lqaf089_Supplemental_File

## Data Availability

The code used for data analysis is available on github: https://github.com/zajacn/scRNAseq_Long_reads_vs_Short_reads; https://zenodo.org/records/15149455. The raw data have been deposited in the European Nucleotide Archive under the accession PRJEB73513 (ERP158282).

## References

[1] Heumos L, Schaar AC, Lance C et al. Best practices for single-cell analysis across modalities. Nat Rev Genet. 2023; 24:550–72. 10.1038/s41576-023-00586-w. 37002403 PMC10066026

[2] Dong X, Tian L, Gouil Q et al. The long and the short of it: unlocking nanopore long-read RNA sequencing data with short-read differential expression analysis tools. NAR Genom Bioinform. 2021; 3:lqab028 10.1093/nargab/lqab028. 33937765 PMC8074342

[3] Cusanovich DA, Daza R, Adey A et al. Multiplex single cell profiling of chromatin accessibility by combinatorial cellular indexing. Science. 2015; 348:910–4. 10.1126/science.aab1601. 25953818 PMC4836442

[4] Buenrostro JD, Wu B, Litzenburger UM et al. Single-cell chromatin accessibility reveals principles of regulatory variation. Nature. 2015; 523:486–90. 10.1038/nature14590. 26083756 PMC4685948

[5] Hao Y, Hao S, Andersen-Nissen E et al. Integrated analysis of multimodal single-cell data. Cell. 2021; 184:3573–87. 10.1016/j.cell.2021.04.048. 34062119 PMC8238499

[6] Stoeckius M, Hafemeister C, Stephenson W et al. Simultaneous epitope and transcriptome measurement in single cells. Nat Methods. 2017; 14:865–8. 10.1038/nmeth.4380. 28759029 PMC5669064

[7] Pai JA, Satpathy AT High-throughput and single-cell T cell receptor sequencing technologies. Nat Methods. 2021; 18:881–92. 10.1038/s41592-021-01201-8. 34282327 PMC9345561

[8] Clark SJ, Argelaguet R, Kapourani C-A et al. scNMT-seq enables joint profiling of chromatin accessibility DNA methylation and transcription in single cells. Nat Commun. 2018; 9:781 10.1038/s41467-018-03149-4. 29472610 PMC5823944

[9] Pott S Simultaneous measurement of chromatin accessibility, DNA methylation, and nucleosome phasing in single cells. eLife. 2017; 6:e23203 10.7554/eLife.23203. 28653622 PMC5487215

[10] Vickovic S, Eraslan G, Salmén F et al. High-definition spatial transcriptomics for *in situ* tissue profiling. Nat Methods. 2019; 16:987–90. 10.1038/s41592-019-0548-y. 31501547 PMC6765407

[11] Yao Z, van Velthoven CTJ, Kunst M et al. A high-resolution transcriptomic and spatial atlas of cell types in the whole mouse brain. Nature. 2023; 624:317–32. 10.1038/s41586-023-06812-z. 38092916 PMC10719114

[12] Panina Y, Karagiannis P, Kurtz A et al. Human cell atlas and cell-type authentication for regenerative medicine. Exp Mol Med. 2020; 52:1443–51. 10.1038/s12276-020-0421-1. 32929224 PMC8080834

[13] Trapnell C Defining cell types and states with single-cell genomics. Genome Res. 2015; 25:1491–8. 10.1101/gr.190595.115. 26430159 PMC4579334

[14] Dann E, Cujba A-M, Oliver AJ et al. Precise identification of cell states altered in disease using healthy single-cell references. Nat Genet. 2023; 55:1998–2008. 10.1038/s41588-023-01523-7. 37828140 PMC10632138

[15] Otto D, Jordan C, Dury B et al. Quantifying cell-state densities in single-cell phenotypic landscapes using Mellon. Nat Methods. 2024; 21:1185–95. 38890426 10.1038/s41592-024-02302-w PMC12265947

[16] Siebert S, Farrell JA, Cazet JF et al. Stem cell differentiation trajectories in *Hydra* resolved at single-cell resolution. Science. 2019; 365:eaav9314. 31346039 10.1126/science.aav9314 PMC7104783

[17] Saelens W, Cannoodt R, Todorov H. et al. A comparison of single-cell trajectory inference methods. Nat Biotechnol. 2019; 37:547–54. 10.1038/s41587-019-0071-9. 30936559

[18] Almet AA, Cang Z, Jin S et al. The landscape of cell–cell communication through single-cell transcriptomics. Curr Opin Syst Biol. 2021; 26:12–23. 10.1016/j.coisb.2021.03.007. 33969247 PMC8104132

[19] Wahiduzzaman , Liu Y, Huang T et al. Cell–cell communication analysis for single-cell RNA sequencing and its applications in carcinogenesis and COVID-19. Biosafety and Health. 2022; 4:220–7. 10.1016/j.bsheal.2022.03.001.

[20] Meng H, Zhang T, Wang Z et al. High-throughput host-microbe single-cell RNA sequencing reveals ferroptosis-associated heterogeneity during *Acinetobacter baumannii* infection. Angew Chem Int Ed Engl. 2024; 63:e202400538 10.1002/anie.202400538. 38419141

[21] Pardo-Palacios FJ, Wang D, Reese F et al. Systematic assessment of long-read RNA-seq methods for transcript identification and quantification. Nat Methods. 2024; 21:1349–63. 10.1038/s41592-024-02298-3. 38849569 PMC11543605

[22] Hård J, Mold JE, Eisfeldt J et al. Long-read whole-genome analysis of human single cells. Nat Commun. 2023; 14:5164 10.1038/s41467-023-40898-3. 37620373 PMC10449900

[23] Dondi A, Lischetti U, Jacob F et al. Detection of isoforms and genomic alterations by high-throughput full-length single-cell RNA sequencing in ovarian cancer. Nat Commun. 2023; 14:7780 10.1038/s41467-023-43387-9. 38012143 PMC10682465

[24] Wu S, Zhang H, Fouladdel S et al. Cellular, transcriptomic and isoform heterogeneity of breast cancer cell line revealed by full-length single-cell RNA sequencing. Comput Struct Biotechnol J. 2020; 18:676–85. 10.1016/j.csbj.2020.03.005. 32257051 PMC7114460

[25] Martinez NM, Lynch KW Control of alternative splicing in immune responses: many regulators, many predictions, much still to learn. Immunol Rev. 2013; 253:216–36. 10.1111/imr.12047. 23550649 PMC3621013

[26] Raj B, Blencowe BJ Alternative splicing in the mammalian nervous system: recent insights into mechanisms and functional roles. Neuron. 2015; 87:14–27. 10.1016/j.neuron.2015.05.004. 26139367

[27] Tian L, Jabbari JS, Thijssen R et al. Comprehensive characterization of single-cell full-length isoforms in human and mouse with long-read sequencing. Genome Biol. 2021; 22:310 10.1186/s13059-021-02525-6. 34763716 PMC8582192

[28] Al’Khafaji AM, Smith JT, Garimella KV et al. High-throughput RNA isoform sequencing using programmable cDNA concatenation. Brief Commun. 2024; 42:582–6. 10.1038/s41587-023-01815-7 PMC12236355 37291427

[29] Cheng O, Ling MH, Wang C et al. Flexiplex: a versatile demultiplexer and search tool for omics data. Bioinformatics. 2024; 40:btae102 10.1093/bioinformatics/btae102. 38379414 PMC10914444

[30] You Y, Prawer YDJ, De Paoli-Iseppi R et al. Identification of cell barcodes from long-read single-cell RNA-seq with BLAZE. Genome Biol. 2023; 24:66 10.1186/s13059-023-02907-y. 37024980 PMC10077662

[31] De Rijk P, Watzeels T, Küçükali F et al. Scywalker: scalable end-to-end data analysis workflow for long-read single-cell transcriptome sequencing. Bioinformatics. 2024; 40:btae549. 39254601 10.1093/bioinformatics/btae549 PMC11419950

[32] Pardo-Palacios FJ, Arzalluz-Luque A, Kondratova L et al. SQANTI3: curation of long-read transcriptomes for accurate identification of known and novel isoforms. Nat Methods. 2024; 21:793–7. 10.1038/s41592-024-02229-2. 38509328 PMC11093726

[33] Tardaguila M, de la Fuente L, Marti C et al. SQANTI: extensive characterization of long-read transcript sequences for quality control in full-length transcriptome identification and quantification. Genome Res. 2018; 28:396–411. 10.1101/gr.222976.117. 29440222 PMC5848618

[34] Bolck HA, Pauli C, Göbel E et al. Cancer sample biobanking at the next level: combining tissue with living cell repositories to promote precision medicine. Front Cell Dev Biol. 2019; 7:246 10.3389/fcell.2019.00246. 31696117 PMC6817465

[35] Bolck HA, Corrò C, Kahraman A et al. Tracing clonal dynamics reveals that two- and three-dimensional patient-derived cell models capture tumor heterogeneity of clear cell renal cell carcinoma. Eur Urol Focus. 2021; 7:152–62. 10.1016/j.euf.2019.06.009. 31266731

[36] Karakulak T, Zajac N, Bolck HA et al. Heterogeneous and novel transcript expression in single cells of patient-derived clear cell renal cell carcinoma organoids. Genome Res. 2025; 35:698–711. 10.1101/gr.279345.124. 40107723 PMC12047245

[37] Zheng GXY, Terry JM, Belgrader P et al. Massively parallel digital transcriptional profiling of single cells. Nat Commun. 2017; 8:698–711. 10.1038/ncomms14049. 28091601 PMC5241818

[38] Lawrence M, Huber W, Pagès H et al. Software for computing and annotating genomic ranges. PLoS Comput Biol. 2013; 9:e1003118 10.1371/journal.pcbi.1003118. 23950696 PMC3738458

[39] Morgan M, Pages H, Obenchain V et al. Rsamtools: binary alignment (BAM), FASTA, variant call (BCF), and tabix file import. 2016; R package https://bioconductor.org/packages/Rsamtools.

[40] Hao Y, Stuart T, Kowalski MH et al. Dictionary learning for integrative, multimodal and scalable single-cell analysis. Nat Biotechnol. 2024; 42:293–304. 10.1038/s41587-023-01767-y. 37231261 PMC10928517

[41] Blanco-Carmona E Generating publication ready visualizations for single cell transcriptomics using SCpubr. bioRxiv1 March 2022, preprint: not peer reviewed 10.1101/2022.02.28.482303.

[42] Li H Minimap2: pairwise alignment for nucleotide sequences. Bioinformatics. 2018; 34:3094–100. 10.1093/bioinformatics/bty191. 29750242 PMC6137996

[43] Andreatta M, Berenstein AJ, Carmona SJ scGate: marker-based purification of cell types from heterogeneous single-cell RNA-seq datasets. Bioinformatics. 2022; 38:2642–4. 10.1093/bioinformatics/btac141. 35258562 PMC9048671

[44] Osorio D, Cai JJ Systematic determination of the mitochondrial proportion in human and mice tissues for single-cell RNA-sequencing data quality control. Bioinformatics. 2021; 37:963–7. 10.1093/bioinformatics/btaa751. 32840568 PMC8599307

[45] Isakova A, Neff N, Quake SR Single-cell quantification of a broad RNA spectrum reveals unique noncoding patterns associated with cell types and states. Proc Natl Acad Sci USA. 2021; 118:e2113568118. 34911763 10.1073/pnas.2113568118 PMC8713755

[46] Benjamini Y, Speed TP Summarizing and correcting the GC content bias in high-throughput sequencing. Nucleic Acids Res. 2012; 40:e72 10.1093/nar/gks001. 22323520 PMC3378858

[47] Zhang Y, Narayanan SP, Mannan R et al. Single-cell analyses of renal cell cancers reveal insights into tumor microenvironment, cell of origin, and therapy response. Proc Natl Acad Sci USA. 2021; 118:e2103240118. 34099557 10.1073/pnas.2103240118 PMC8214680

[48] Young MD, Mitchell TJ, Vieira Braga FA et al. Single-cell transcriptomes from human kidneys reveal the cellular identity of renal tumors. Science. 2018; 361:594–9. 10.1126/science.aat1699. 30093597 PMC6104812

[49] Bi K, He MX, Bakouny Z et al. Tumor and immune reprogramming during immunotherapy in advanced renal cell carcinoma. Cancer Cell. 2021; 39:649–61. 10.1016/j.ccell.2021.02.015. 33711272 PMC8115394

[50] Chen Z, Han F, Du Y et al. Hypoxic microenvironment in cancer: molecular mechanisms and therapeutic interventions. Signal Transduct Target Ther. 2023; 8:70 10.1038/s41392-023-01332-8. 36797231 PMC9935926

[51] Miikkulainen P, Högel H, Seyednasrollah F et al. Hypoxia-inducible factor (HIF)-prolyl hydroxylase 3 (PHD3) maintains high HIF2A mRNA levels in clear cell renal cell carcinoma. J Biol Chem. 2019; 294:3760–71. 10.1074/jbc.RA118.004902. 30617181 PMC6416423

[52] Schutgens F, Rookmaaker MB, Margaritis T et al. Tubuloids derived from human adult kidney and urine for personalized disease modeling. Nat Biotechnol. 2019; 37:303–13. 10.1038/s41587-019-0048-8. 30833775

[53] Deng E, Shen Q, Zhang J et al. Systematic evaluation of single-cell RNA-seq analyses performance based on long-read sequencing platforms. J Adv Res. 2025; 71:141–53. 10.1016/j.jare.2024.05.020. 38782298 PMC12126699

[54] Macosko EZ, Basu A, Satija R et al. Highly parallel genome-wide expression profiling of individual cells using nanoliter droplets. Cell. 2015; 161:1202–14. 10.1016/j.cell.2015.05.002. 26000488 PMC4481139

[55] Ilicic T, Kim JK, Kolodziejczyk AA et al. Classification of low quality cells from single-cell RNA-seq data. Genome Biol. 2016; 17:29 10.1186/s13059-016-0888-1. 26887813 PMC4758103

[56] Ordoñez-Rueda D, Baying B, Pavlinic D et al. Apoptotic cell exclusion and bias-free single-cell selection are important quality control requirements for successful single-cell sequencing applications. Cytometry A. 2020; 97:156–67. 10.1002/cyto.a.23898. 31603610

[57] Lun ATL, Riesenfeld S, Andrews T et al. EmptyDrops: distinguishing cells from empty droplets in droplet-based single-cell RNA sequencing data. Genome Biol. 2019; 20:63 10.1186/s13059-019-1662-y. 30902100 PMC6431044

[58] Muskovic W, Powell JE DropletQC: improved identification of empty droplets and damaged cells in single-cell RNA-seq data. Genome Biol. 2021; 22:329. 34857027 10.1186/s13059-021-02547-0 PMC8641258

[59] Garratt LW Current understanding of the neutrophil transcriptome in health and disease. Cells. 2021; 10:2406 10.3390/cells10092406. 34572056 PMC8469435

[60] La Manno G, Soldatov R, Zeisel A et al. RNA velocity of single cells. Nature. 2018; 560:494–8. 10.1038/s41586-018-0414-6. 30089906 PMC6130801

[61] Feng S, Xu M, Liu F et al. Reconstruction of the full-length transcriptome atlas using PacBio Iso-Seq provides insight into the alternative splicing in *Gossypium australe*. BMC Plant Biol. 2019; 19:365 10.1186/s12870-019-1968-7. 31426739 PMC6701088

[62] Clark TA Single molecule, real-time sequencing of full-length cDNA transcripts uncovers novel alternatively spliced isoforms. 2015; (22 June 2025, date lastaccessed) https://www.pacb.com/wp-content/uploads/Single-Molecule-Real-Time-Sequencing-of-Full-length-cDNA-Transcripts-Uncovers-Novel-Alternatively-Spliced-Isoforms.pdf.

[63] Ali A, Thorgaard GH, Salem M PacBio Iso-Seq improves the rainbow trout genome annotation and identifies alternative splicing associated with economically important phenotypes. Front Genet. 2021; 12:683408 10.3389/fgene.2021.683408. 34335690 PMC8321248

[64] Suslov O, Silver DJ, Siebzehnrubl FA et al. Application of an RNA amplification method for reliable single-cell transcriptome analysis. Biotechniques. 2015; 59:137–48. 10.2144/000114331. 26345506 PMC4832400

[65] Apavaloaei A, Hardy M-P, Thibault P et al. The origin and immune recognition of tumor-specific antigens. Cancers. 2020; 12:2607. 32932620 10.3390/cancers12092607 PMC7565792

[66] Camarena ME, Theunissen P, Ruiz M et al. Non-canonical ORFs are an important source of tumor-specific antigens in a liver cancer meta-cohort. bioRxiv1 November 2023, preprint: not peer reviewed 10.1101/2023.10.30.564375.

[67] Abugessaisa I, Noguchi S, Hasegawa A et al. RefTSS: a reference data set for human and mouse transcription start sites. J Mol Biol. 2019; 431:2407–22. 10.1016/j.jmb.2019.04.045. 31075273

