# Supplementary material for: Comparison of single-cell long-read and short-read transcriptome sequencing via cDNA molecule matching: quality evaluation of the MAS-ISO-seq approach
Source: NAR Genom Bioinform. 2025 Jul 4;7(3):lqaf089. doi: 10.1093/nargab/lqaf089 (PMC12231600; doi:10.1093/nargab/lqaf089)
Supplement: lqaf089_Supplemental_File [file lqaf089_supplemental_file.docx]

Supplementary Figures for «Comparison of Single-cell Long-read and Short-read Transcriptome Sequencing via cDNA molecule matching: Quality Evaluation of the MAS-ISO-seq Approach »

Content:

1. Figure S1. Barcode Rank Plots for samples Normal, ccRCC_2, ccRCC_4 and ccRCC_5. The plots show high RNA content cells found both in PacBio and Illumina data and the low RNA content cells found uniquely in Illumina data.
2. Figure S2. Venn diagrams showing the sharing of cell barcodes identified as real cells by CellRanger in Illumina data (ILL) with cell barcodes identified as real cells by Iso-Seq in PacBio (PB) data and cell barcodes identified as empty droplets by BLAZE in PacBio data. Percentages indicate the proportion of cell associated barcodes from CellRanger.
3. Figure S3. Mapping summary of subsampled short-read and long-read sequencing data by cell barcode IDs beginning with GC (13-59 cells) or TG (21-85 cells). The data for barcodes beginning with AA (15-48 cells) is summarised within the main text. a.) Number of common and unique combinations of cell barcode + UMI considered in the comparison of bam files (mapped data) between PacBio and Illumina. b.) Fraction of PacBio reads shared with Illumina represented across different read length categories (log2 of read length, 7-11). c.) Sankey plot visualising the proportion of tag IDs shared between Illumina and PacBio that are uniquely mapping/multi mapping, map to the same location in the genome or to a different location, are counted or not into the gene count matrix and the types of PacBio-specific artefacts they belong to when not counted. d.) The proportion of TSO and polyA contamination in reads unique to Illumina and Illumina reads shared with PacBio. e.) Logged abundance of cell barcode-UMI tags across different types of PacBio-specific and Illumina-specific annotations. The unannotated reads from PacBio are reads discarded due to mapping chimerically or mapping with poor identity. Illumina additionally has a set of reads that were unmapped (but were mapped by PacBio).
4. Figure S4. Pearson correlation between PacBio (unfiltered of any isoforms) and Illumina data in the log-mean normalised sum of counts per gene across all common cells coloured by GC content and gene length category in all samples.
5. Figure S5. Gene length distribution of genes with significantly higher counts in Illumina (edgeR exactTest, logFC > 1, FDR < 0.05), with counts equal between Illumina and PacBio and with significantly higher counts in PacBio (edgeR exactTest, logFC < 1, FDR < 0.05). The PacBio data used was unfiltered of any isoforms. T-test in was used to compare the significance of the difference between the different groups.
6. Figure S6. GC content distribution of genes with significantly higher counts in Illumina (edgeR exactTest, logFC > 1, FDR < 0.05), with counts equal between Illumina and PacBio and with significantly higher counts in PacBio (edgeR exactTest, logFC < 1, FDR < 0.05). The PacBio data used was unfiltered of any isoforms. T-test was used to compare the significance of the difference between the 3 groups.
7. Figure S7. UMAP embeddings for all samples, coloured by sequencing method. Bottom: bar plots representing the number of cells from each method in each cluster.
8. Figure S8. Pearson correlation between PacBio data filtered of isoforms and PacBio unfiltered data in the sum of counts per gene across all common cells coloured by GC content and gene length category across all samples.
9. Figure S9. UMAP embeddings for all samples, coloured by sequencing method. Here PacBio data is filtered of all artefactual isoforms. Bottom: bar plots representing the number of cells from each method in each cluster.

Illumina Specific Cells

Illumina Specific Cells

PacBio Common Cells

PacBio Common Cells

Illumina Common Cells

Illumina Common Cells

Illumina Specific Cells

Illumina Specific Cells

PacBio Common Cells

PacBio Common Cells

Illumina Common Cells

Illumina Common Cells

Figure S1.


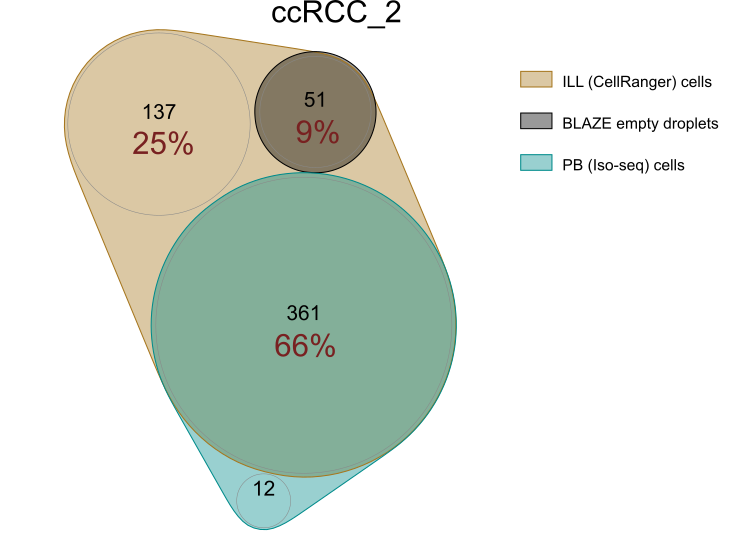

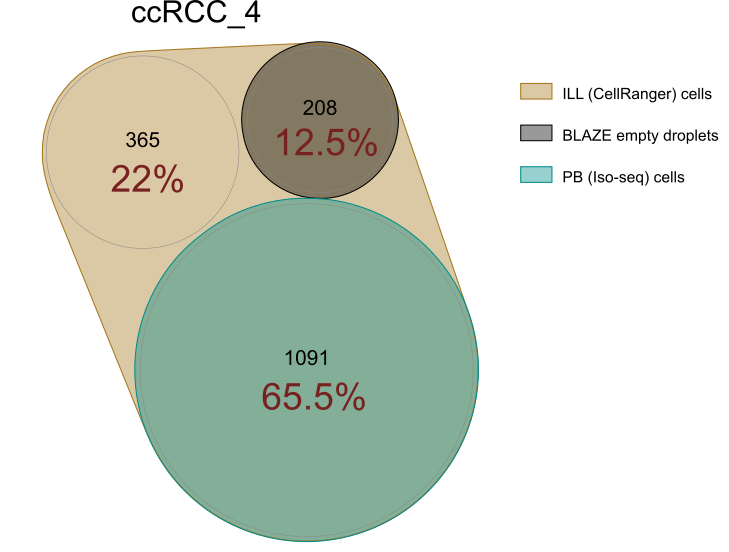


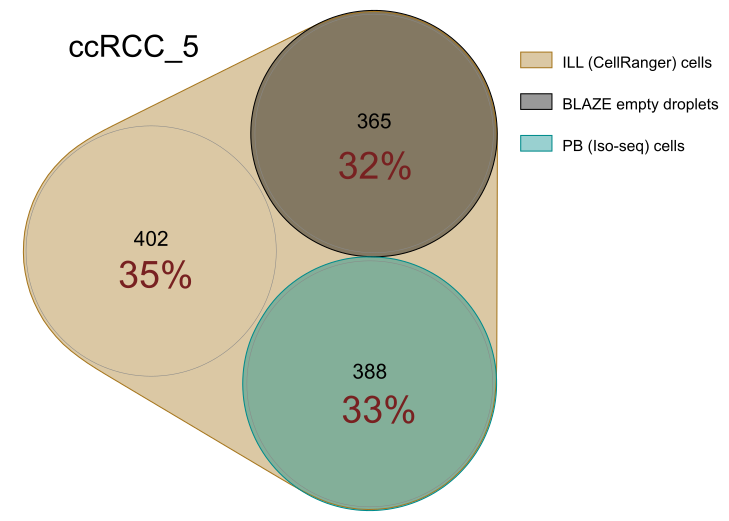

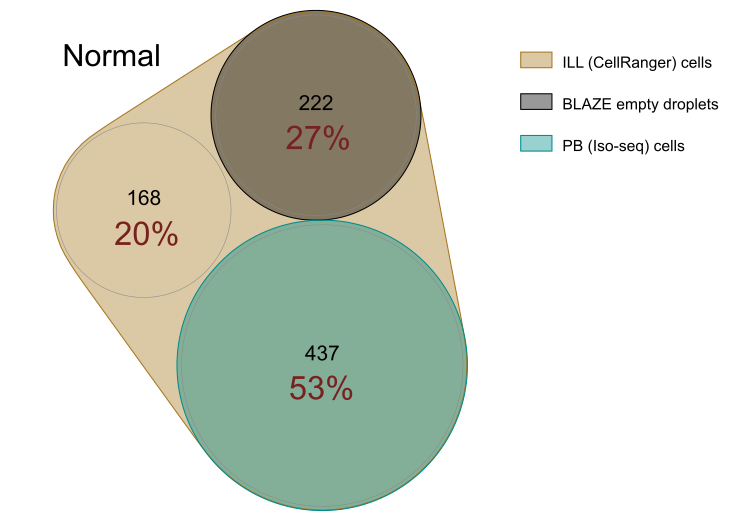


Figure S2.

B.

A.

Subsampled-GC

Subsampled-TG

Subsampled-GC

Subsampled-TG

C.

D.

Subsampled-GC

Subsampled-TG

Subsampled-TG

E.

Subsampled-TG

Subsampled-GC

Subsampled-TG

Subsampled-GC

Figure S3.

 Figure S4.

Figure S5.

Figure S6.

 Figure S7.

Figure S8.

Figure S9.
